# Relationship between the Presence of the nalC Mutation and Multidrug Resistance in *Pseudomonas aeruginosa*

**DOI:** 10.1155/2012/575193

**Published:** 2012-05-07

**Authors:** Nourkhoda Sadeghifard, Azar Valizadeh, Mohammad Reza Zolfaghary, Mohammad Hossien Maleki, Abbas Maleki, Reza Mohebi, Sobhan Ghafourian, Afra Khosravi

**Affiliations:** ^1^Clinical Microbiology Research Center, Ilam University of Medical Sciences, Ilam 693917134, Iran; ^2^Department of Microbiology, Qom Branch, Islamic Azad University of Qom, Qom, Iran

## Abstract

*Objectives*. The current study was conducted to determine the relationship between the presences of significant multidrug resistance in *Pseudomonas aeruginosa *(*P. aeruginosa*) having intact *mexR *genes (nalC) to different antibiotics. *Methods*. In order to identify nalC, fifty strains of *P. aeruginosa *were obtained. All isolates were found in urinary tract infections. They were evaluated against different antibiotics. The nalC mutant was identified by PCR. *Results*. The 50 clinical isolates of *P. aeruginosa *originated from two hospitals in Iran, in which 32 isolates were found in Milad hospital, and 18 isolates were collected in the Ilam Hospital. The results in Milad hospital of nalC revealed that all *P. aeruginosa *resistant to oxacillin showed the presence of nalC. In Ilam hospital only three (16.6%) isolates were resistant to oxacilin and aztreonam, and among these three isolates only one isolate revealed resistance to ceftazidime and amikacin. The resistant isolates showed the presence of both OXA-10 and nalC. *Conclusion*. Our results showed that the presence of nalC was observed among *P. aeruginosa *resistance to oxacilin. Thus, the finding suggested relationship between oxacilin resistance and presence of nalC and consequently overproduction of the MexABOprM efflux system.

## 1. Introduction

*P. aeruginosa *is an opportunistic human pathogen characterized by an innate resistance to multiple antimicrobials [1]. *P. aeruginosa* is responsible for 10–15% of the nosocomial infections worldwide [2]. Often these infections are hard to treat due to the natural resistance of the species as well as to its remarkable ability of acquiring further mechanisms of resistance to multiple groups of antimicrobial agents.

Oxacillinases (OXA-type enzymes) belong to molecular class D and functional group 2 [3]. Classical OXA enzymes (OXA-1, OXA-2, and OXA-10) determine resistance to carboxypenicillins and ureidopenicillins but not to ceftazidime [4]. Resistance to ticarcillin and piperacillin resulting from production of OXA-2 enzymes is lower than the resistance that develops when OXA-10 and OXA-1 oxacillinases are produced [4]. Generally, *P. aeruginosa* clinical isolates are less susceptible than Enterobacteriaceae to most classes of antimicrobials. For a long time the main reason for this natural resistance was considered to be the low outer membrane permeability due to the presence of proteins with high molecular mass about 50 kDa [5].

Efflux contributes to the development of multiple resistances to all strategic antipseudomonal antibiotics and is mediated by four genetically different three component efflux systems that belong to the resistance-nodulation-division (RND) family: MexA-MexB-OprM, MexC-MexD-OprJ, MexE-MexF-OprN, and MexX-MexY-OprM. The first component is a protein located in the cytoplasmic membrane (MexB, MexD, MexF, and MexY) that operates as an energydependent pump with wide substrate specificity. The second component is a gated outer membrane protein (OprM, OprJ, OprN, and OprM). The third protein (MexA, MexC, MexE, and MexX) is located in the periplasmic space and links the other two [6].

There are also other mutants called nalC that have intact mexR genes [7]. nalC mutants originate from the wild-type *P. aeruginosa* PAO1 and are characterized by a mutation in the PA3721 gene [8]. The protein encoded by this gene is a repressor of a two-gene operon; its function is unclear, and its overexpression in nalC mutants leads to overproduction of the MexAB-OprM efflux system.

The current study was conducted to determine the relationship between the presence of the nalC mutation in *P. aeruginosa* and its resistance to antibiotics.

## 2. Material and Methods

### 2.1. Bacterial Strains

 In order to identify nalC, fifty strains of *Pseudomonas aeruginosa* obtained during the period of March 2010 to August 2010 in the Milad and Ilam hospitals, from which 32 isolates were from the Milad hospital, and 18 isolates were from the Ilam hospital. All isolates were isolated from urinary tract infections.

*P. aeruginosa* which was resistant to *cefotaxime*, *ceftazidime*,* amikacin, oxacillin, aztreonam, and ticarcillin* was ESBLs positive, and the presence of OXA-2 and OXA-10 was assayed by the PCR for detection of ESBLs genes.

### 2.2. PCR

PCR was performed for detection of OXA-10 and OXA-2 genes. The primers used were as follows:

OXA-2 gene; Forward 5-GCC AAA GGC ACG ATA GTT GT-3;
Reverse 5-GCG TCC GAG TTG ACT GCC GG-3;
OXA-10 gene; Forward: 5-GT CTT TCG AGT ACG GCA TTA-3;
Reverse 5-ATT TTC TTA GCG GCA ACT TAC-3.

### 2.3. PCR for Detection of NalC

NalC was identified by using of Forward: 5-TCA ACC CTA ACG AGA AAC GCT-3 and

Reverse 5-TCC ACC TCA CCG AAC TGC-3.

## 3. Results

The 50 clinical isolates of *P. aeruginosa* originated from two hospitals in Iran, which from 32 isolates were collected in the Milad hospital, and 18 isolates collected in the Ilam Hospital. After the identification of isolates as *P. aeruginosa,* they were evaluated for susceptibility to different antibiotics. The finding in Milad hospital revealed that 87.1% (*n* = 25) of the isolates that expressed resistance to oxacilin and resistance to *cefotaxime*, *ceftazidime*,* amikacin, aztreonam, and Ticarcillin* were 62.5% (*n* = 20), 50% (*n* = 16), 56.25% (*n* = 18), and 68.75% (*n* = 22), respectively. The findings showed that all oxacilin resistant strains harbored OXA-10, while only 2 isolates showed OXA-2 genes. Therefore, two isolates harbored both OXA-10 and OXA-2. The results of determining nalC revealed that all *P. aeruginosa* resistant to oxacilin showed the presence of nalC. Therefore, 87.1% of *P. aeruginosa* harbored nalC, in addition, 7 *P. aeruginosa* which were negative for nalC also were susceptible to the others antibiotics. There was a significant difference observed between the presence of nalC and oxacilin resistant strains. In Ilam hospital only three (16.6%) isolates were resistant to oxacilin and aztreonam, and among these three isolates only one isolate was resistant to ceftazidime and amikacin. The resistant isolates showed the presence of both OXA-10 and nalC. The findings showed that no nalC was observed in sensitive isolates in both hospitals. A significant difference was observed among resistant strains to oxacilin and the presence of nalC, while no significant difference occurred among resistance strains to the other antibiotics and nalC.

## 4. Discussion

*P. aeruginosa *presents a therapeutic challenge for treatment of both community-acquired and nosocomial infections. Unfortunately, selection of the most appropriate antibiotic is complicated by the ability of *P. aeruginosa *to develop resistance to multiple classes of antibacterial agents, even during the course of treating an infection [9]. Epidemiological outcome studies have shown that infections caused by drug-resistant *P. aeruginosa *are associated with significant increases in morbidity, mortality, the need for surgical intervention, the length of hospital stay and chronic care, and the overall cost of treating the infection [10].

Resistance to ticarcillin and piperacillin resulting from the production of OXA-2 enzymes is lower than the resistance that develops when OXA-10 and OXA-1 oxacillinases are produced [4]. The results of this study showed that OXA-2 enzyme was found in two isolates which demonstrated resistance to ticarcillin.

The results showed a significant difference between nalC and oxacilin resistant strains.

MexA-MexB-OprM overproduction often occurs in clinical isolates of *P. aeruginosa,* and usually it is a result of increased transcription of the mexA-mexB-oprM operon due to mutations in the chromosomal gene encoding the MexR repressor protein, that is, mutations at the mexR locus [11]. The protein encoded by this gene is a repressor of a two-gene operon; its function is unclear, and its overexpression in nalC mutants leads to overproduction of the MexAB-OprM efflux system.

In conclusion, Nalc exists as an important factor in the increased expression of pumps MEXAB-OPRM, started its presence in samples containing ESBL has been reported in the world. Several reasons can explain the relationship between nalc and beta-lactamase such as the effects of creation of nalc, is the increase of expression of porin MEX AB-OPRM. Our results showed that the presence of nalC occurred concomitant with resistance to oxacilin. Thus, the finding suggested a relationship between oxacilin resistance and the presence of nalC and consequently overproduction of the MexAB-OprM efflux system. Our results revealed that more study is needed for finding a way which is able to reduce the oxacilin resistance and lead to reduction of production of MexAB-OprM efflux system.
